# Dr. William Rice Pryor

**Published:** 1904-10

**Authors:** 


Dr. William Rice Pryor, of New York, died at Saint Vincent's Hospital in that city, August 25, 1904, aged 46 years. Dr. Pryor had attained eminence as a gynecological surgeon and was the author of many articles relating to his special line of work, as well as a textbook on gynecology. He was the youngest son of Roger A. Pryor, a former brigadier general in the Confederate service during the civil war, and a former judge of the Supreme Court in New York.

Dr. Pryor was graduated from the College of Physicians and Surgeons in 1881 and became professor of gynecology in the New York Polyclinic in 1884. He was visiting gynecologist to the Polyclinic Hospital and consultant at St. Vincent's. He was famous for his considerate attention to the poor, whom he served with a wise liberality, even beyond his means. What better epitaph could be inscribed on a tomb?

